# Efficient Star Identification Using a Neural Network

**DOI:** 10.3390/s20133684

**Published:** 2020-06-30

**Authors:** David Rijlaarsdam, Hamza Yous, Jonathan Byrne, Davide Oddenino, Gianluca Furano, David Moloney

**Affiliations:** 1 Intel Corporation, Intel R&D Ireland Ltd, Collinstown, Collinstown Industrial Park, Co., Kildare W23 CX68, Ireland; hamza.yous@intel.com (H.Y.); jonathan.byrne@intel.com (J.B.); david.moloney@intel.com (D.M.); 2 European Space Agency/ESTEC, Keplerlaan 1, 2201 AZ Noordwijk, the Netherlands; davide.oddenino@esa.int (D.O.); gianluca.furano@esa.int (G.F.)

**Keywords:** star identification, deep learning, lost-in-space, star feature extraction

## Abstract

The required precision for attitude determination in spacecraft is increasing, providing a need for more accurate attitude determination sensors. The star sensor or star tracker provides unmatched arc-second precision and with the rise of micro satellites these sensors are becoming smaller, faster and more efficient. The most critical component in the star sensor system is the lost-in-space star identification algorithm which identifies stars in a *scene* without a priori attitude information. In this paper, we present an efficient lost-in-space star identification algorithm using a neural network and a robust and novel feature extraction method. Since a neural network implicitly stores the patterns associated with a guide star, a database lookup is eliminated from the matching process. The search time is therefore not influenced by the number of patterns stored in the network, making it constant (O(1)). This search time is unrivalled by other star identification algorithms. The presented algorithm provides excellent performance in a simple and lightweight design, making neural networks the preferred choice for star identification algorithms.

## 1. Introduction

With the requirements on attitude determination for spacecraft becoming more strict, a need for more accurate attitude sensors is apparent. Star sensors or star trackers are the most accurate sensors currently on the market, achieving arc-second precision. The star sensor takes an image of the celestial sky and uses the information in that *scene* to uniquely identify the stars present. The attitude is then determined using a look-up table and a attitude determination algorithm. The most critical component of the star sensor is the lost-in-space identification algorithm. This algorithm has as input the centroid locations and magnitudes of the light sources present in the scene and identifies the stars present without a priori attitude information. Many examples of these algorithms can be found in literature, which can be classified by the employed feature extraction method. Two categories are defined: pattern- and subgraph isomorphism-based feature extraction [1]. The pattern feature extraction algorithms assign each guide star a pattern based on the stars surrounding it and tries to find the closest matching pattern in a database. Examples of these algorithms include the grid algorithm [2], the singular value method algorithm [3] and the Log-Polar algorithm [4]. The subgraph isomorphism feature extraction algorithms treat the stars as vertices in a subgraph and the angular distances between the stars as edge weights. These algorithms try to find the relevant isomorphic subgraph in a database. Examples are triangle algorithms [5], group match algorithms [6] and the pyramid algorithm [7]. In this paper, a pattern feature extraction method is presented.

Deep learning approaches for star identification algorithm are appealing, since they eliminate the otherwise required database search and therefore are able to provide an analytical performance of O(1), i.e., The search time is constant and independent of the number of patterns stored in the network. Therefore, the analytical performance of a deep learning approach is unrivalled by other star identification algorithms [1]. Neural networks are not new to the field of star identification algorithms. In 1989 the first proposal on using neural networks was published [8,9]. Hong and Dickerson published a neural network based star identification algorithm in 2000 [10]. The parallel hardware needed to run inference using a neural network was not space-graded at the time [9]. Due to the performance improvements in deep learning and the availability of more parallel hardware, this is no longer an issue in the near future. Jing and Liang showed that a neural network can be used to recognise classical feature patterns produced by the grid algorithm [11]. However, their algorithm required multiple subnets and a coarse identification which made the solution highly complex. More recently, work by Xu et al. has included a star identification algorithm that uses a combination of a pattern generator and a classifier called RPnet [12]. While this algorithm showed promise, it is complex with 4 fully connected layers and may be limited to larger field of view (FOV) sensors.

In this paper, we shall present a novel and efficient method to identify stars in a scene using a neural network, returning the star label. The work presented here is focused on the feature extraction of a star scene and the classification of this star scene, but does not include a verification step. In future work, we shall implement this solution in an end-to-end lost-in-space star identification algorithm. We show in this paper that it is possible to use a relatively simple and small fully connected network for star identification as long as the feature extraction from the scene is performed appropriately. The network is able to classify a large number of stars and can be implemented on existing high Technology Readiness Level (TRL) hardware. It is not prohibitively large, solving the existing issues with using deep learning for star identification. Furthermore, because the star patterns are implicitly saved in the network the time expensive database search necessary for classical star identification algorithms is removed entirely from the process. Due to the robust feature extraction method, the algorithm is very resilient to noise. Therefore the contribution of this work is two-fold: a novel and efficient way to perform pattern based feature extraction and an efficient neural network based state-of-the-art identification algorithm. We shall first describe our method by covering the novel feature extraction approach and our neural network architecture. Then we go into the performance results found using a simulation test. Finally, we provide a discussion including recommendations for future work.

## 2. Method

A star identification algorithm needs to be fast, accurate, resource efficient, reliable and should have low complexity. Naturally a trade-off exists between these characteristics. However, as stated above, in terms of analytical performance deep learning based star identification algorithms provide unrivalled performance compared to all other star identification algorithms [1]. Since a neural network can achieve O(1) search time, it is an obvious choice to apply to star identification.

In current publications on deep learning approaches for star identification algorithms this trade-off between accuracy, complexity and resource use remains: a more accurate network uses more complex architectures that require a larger amount of computing resources. We address this by using a powerful but simple feature extraction method. This method can be used as input to a small, accurate and efficient neural network architecture. This network has the analytical performance of a deep learning solution while maintaining accuracy, low complexity, low resource use and high robustness against noise.

In a star sensor system, the star identification algorithm is preceded by a star centroiding algorithm that provides sub-pixel accuracy on the centroid determination of stars present in a scene. A convolutional neural network would lose this detailed information due to the applied convolutions. Therefore, in order to maintain this accuracy, a fully connected architecture was designed that uses the highly accurate centroids provided by the centroiding algorithm.

We shall present both the feature extraction method and the applied architecture in this section.

### 2.1. Novel Feature Extraction

A neural network requires an input of invariant size and order, i.e., it is necessary to use an order- and rotational-invariant feature extraction. In order to create such a pattern, multiple approaches are possible. For example, the approach used by the classical Grid Algorithm is to rotate the pattern to a predefined orientation using two reference stars [2]. Obviously, this makes the pattern very sensitive to noise due to the probability of choosing the wrong star as reference. According to [13], the probability of choosing the right reference star can be as low as 50%. Another approach is to use a polestar-based pattern. First proposed by Silani and Lovera in 2006, the Polestar algorithm uses the rotational invariant binned distances to the pattern stars from a centered polestar [14]. This pattern is used to select candidate stars, after which Silani and Lovera used a subgraph isomorphism based feature extraction step to uniquely identify the polestar. The binned distances are rotational invariant and the order of features is constant making an adaption of this feature extraction applicable to star identification using a neural network.

**Figure 1:**
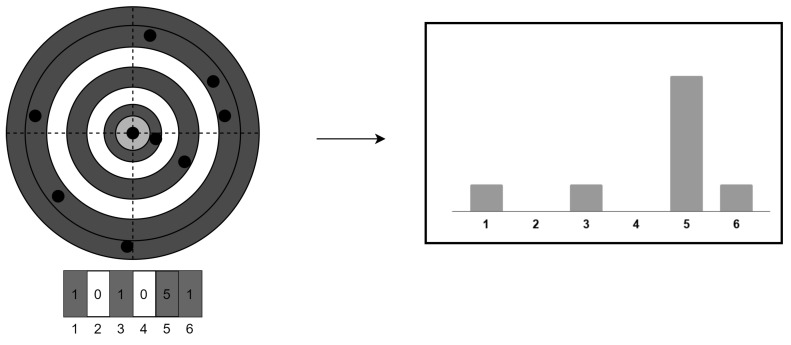
Feature extraction method.

In order to reduce the variance and improve the performance of the neural network, we create a histogram of the binned distances as input to the network (Figure 1). The input to the network is the distribution of distance to the polestar, with the sum of the values of the bins being equal to 1. While this pattern only retains one-dimensional spatial information, we shall show that this is sufficient to achieve excellent performance and that the pattern provides the added benefit of being resilient to false stars. This is because a relatively large number of stars is needed to disturb the one-dimensional distribution.

A number of hyperparameters which depend on the application environment can be defined when using this feature extraction method. The number of bins $n_{bins}$ should be determined using simulation of the application environment. The bin distances $d_{i}$ where $i=1,2,\ldots ,n_{bins}$ should be spaced optimally: in a real-world application the bins closer to the guide star need to be smaller than the bins further away as the probability of information (other stars) being present quadratically decreases as the distance to the guide star increases. This is due to the fact that the guide star needs to be translated to the center of the scene if it is not captured at that position. This causes information at the edges of the pattern to be missing since that information was not captured in the image. We shall cover this in future work and use equally distanced bins here ($d_{i}=c$ for $i=1,2,\ldots ,n_{bins}$).

### 2.2. Neural Network Architecture

**Figure 2:**
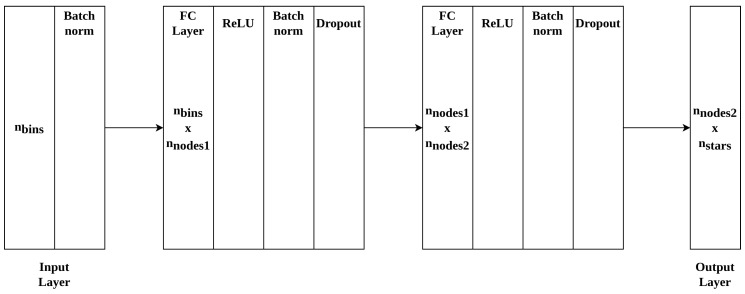
Proposed neural network for lost-in-space star identification. Layer sizes are variable and dependant on application environment.

The required low complexity and small size restricts the possible choices for a neural network architecture for this problem. We have reduced the problem to tabular pattern recognition with our novel feature extraction method. Since no function can be approximated that would allow regression on this problem, we consider the star identification problem as we present it a tabular classification problem. For this classification task, the network architecture is as shown in Figure 2.

The input layer size is dependant on $n_{bins}$, while the optimal two fully connected layer sizes $n_{nodes1}$ and $n_{nodes2}$ are determined experimentally. The number of nodes in the output layer depends on the application environment: a larger number of classes $n_{stars}$ requires a larger number of nodes per layer. The sensitivity of the network performance to number of nodes is shown in Section 3. A class in this model is a star that is identifiable by the network. It should be noted that the architecture presented here is universally applicable to different application environments and can be scaled up accordingly, we therefore do not specify a fixed layer size. After the input layer we use a batch normalisation layer and after both fully connected layers a non-linearity (ReLU) [15], batch normalisation [16] and a dropout layer [17] are applied.

### 2.3. Training

**Figure 3:**
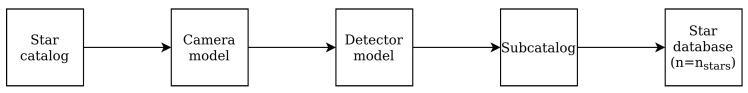
Database generation.

In order to train the network, training data has to be collected. Since multiple open-source catalogues of stars present on the celestial sphere are available, this data is easily generated. Using a straightforward camera and detector model, limitless amounts of tabular data can be generated. Because the model needs to deal with any arbitrary rotation of the pattern, multiple scenes need to be generated for one class. From an existing star catalog a subsection is taken based on a model of the camera and detector used in the sensor (e.g., stars that are too dim to be detected by the detector are excluded), displayed in Figure 3.

From the subcatalog a selection of stars is made that we define as the star database. The star database consists of $n_{stars}$ that the algorithm needs to be able to classify. $n_{stars}$ can be equal to or lower than the size of the subcatalog. The selection criteria for $n_{stars}$ depend on factors such as the FOV of the system, the detector sensitivity, *double stars* (stars that are too closely spaced to accurately determine the centroid) and the required celestial sphere coverage.

**Figure 4:**
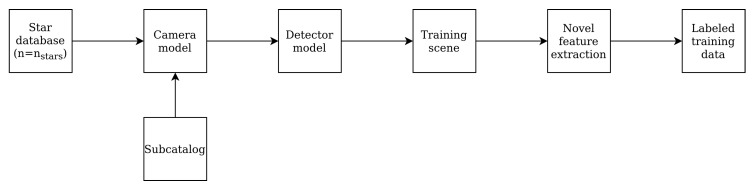
Training data generation.

Training data is generated by looping over all guide stars in the database. For these stars, a scene is generated with the guide star in the center and the stars within the specified FOV present in the subcatalog (Figure 4).

The camera and detector model are used to generate realistic scenes, and include relevant levels of positional noise, magnitude noise, false stars and dropped stars. For each guide star, a number scenes with different rotations is generated in order to teach the network rotational variations. A scene consists of an array of x,y coordinates of centroids present in the image frame.

From a scene the novel feature extraction method is used to generate the labelled training data. The label is the star id number corresponding to the index of the guide star in the star database. In the training of the model, Flattened Cross Entropy is used as the loss function. The model is trained using weight decay [18] to improve generalisation. A common training approach is used that employs gradient descent and uses backpropagation to flow back information of the cost to calculate the gradient, as described by Goodfellow et al. [19]. The number of epochs and learning rate for training the model depend again on the application environment, model size and amount of training data and are found experimentally for the applied setup.

## 3. Results

In order to test the underlying robustness of the star identification algorithm to different levels of noise, a structured testing approach was applied. While this approach does not show the absolute best performance of the star identification algorithm, it does show how the algorithm behaves under different levels of noise. In this section we present the results of these tests and show the underlying robustness of our star identification algorithm against false stars and positional noise. Furthermore, we show the effect of shrinking the network size on the identification rate of the model.

### 3.1. Experimental Setup

The experimental setup used to test the star identification algorithm for underlying robustness consists of an input of binned and labelled star scenes with a star in the center of the image and a pre-trained neural network from which the output is compared to the ground truth. The simulation model for generating these test scenes directly gives the centroid of the stars present in the image, which removes the influence of the centroiding algorithm from the performance measurements. The used networks have been trained on the same dataset, generated using the Hipparcos catalog [20]. This set has 360 scenes per catalog star, under 360 different rotations and with randomly added noise. The relevant parameters for this dataset can be found in Table 1. The number of bins has been chosen to be 25, based on a trade-off between information retention and network size. The optimal value of $n_{bins}$ depends on the FOV and cut-off magnitude threshold as well as the noise environment. For the given dataset, we found that the value of $n_{bins}$ shown here is appropriate. Nevertheless, the sensitivity of the performance of the network to this hyperparameter needs to be investigated in future work.

We measure the performance of the network in terms of *identification rate*, which we define as the number of correct identifications in the test date over the number of incorrect identifications. Furthermore, we record the number of uniquely misidentified stars. This is the number of stars with a different label that have been wrongly identified and shows whether or not the feature extraction method provides uniformly identifiable patterns among the different guide stars.

### 3.2. Robustness to False Stars

In the application of star sensor systems, false stars are part of the input to the system. These false stars can be reflecting dust particles, real stars that are not present in the star database, other spacecraft etc. The star identification algorithm needs to be able to deal with this kind of noise in order to be robust in more demanding application environments. In order to test this robustness, we test the network in steps using an increasing amount of false star percentage in each step. Since the number of nodes in the neural network directly influences the amount of information it can store, the question of what the optimal size of the network is in terms of nodes in both hidden layers arises. In order to find a qualitative answer to this question, the false star experiment was conducted with two network sizes: one with 4096 nodes in both hidden layers, and one with 32 nodes in the first layer and 64 nodes in the second layer respectfully. Both networks have been trained to recognise 2306 classes on the presented data set. The networks were tested also on the same dataset, for which the parameters can be found in Table 2. Note that all camera and detector parameters are the same as the test dataset.

**Figure 5:**
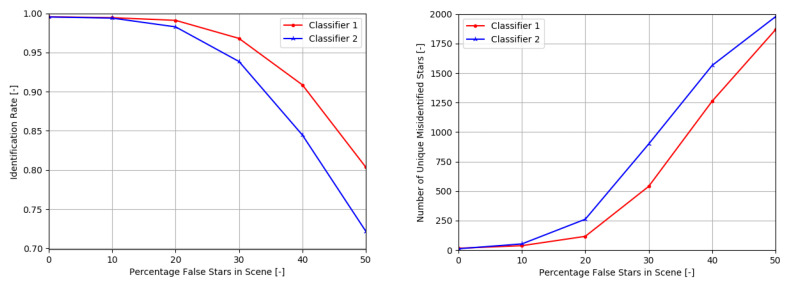
Identification rate of two classifiers under influence of false stars. Classifier 1 has 4096 nodes in both hidden layers, Classifier 2 has 32 in the first hidden layer and 64 nodes in the second hidden layer, respectfully.

The influence of false stars on the performance of both networks can be seen in Figure 5. Clearly, the larger network performs better than the smaller network. However, the difference in performance is most noticeable under more extreme application environments: if up to 10% of false stars is added to a scene, the difference in performance between both networks is negligible. While this performance is not the same as the performance of this algorithm applied in an end-to-end star identification algorithm (where not every test scene would have a star in the center of the scene), it shows that the underlying robustness of the feature extraction method and neural network against false stars is excellent.

The number of uniquely misidentified stars under the influence of false stars is also shown in Figure 5. The number of uniquely misidentified stars grows faster with the smaller network as the noise percentage increases. This indicates that the smaller network is less able to distinguish the unique star patterns under the influence of noise. However, even in an environment with 30% false stars, more than 50% of the stars in the experiment are always correctly recognised for both networks.

While the optimal size of the network depends on the application environment, this experiment shows that even an extremely small network is able to retain enough information for adequate star identification under influence of noise. This results also implies that the larger network is able to retain many more guide stars than are currently in the catalog, although this remains to be verified in future work. Note that other factors that may influence network size are the number of bins used as input to the network and the number of classes needed in the output layer.

The robustness against false stars is remarkable, since the training set used to train these networks included between 0 and 4 false stars per image, much less then the environments tested here. This implies that the performance could be improved by increasing the size of the training dataset with a more diverse noise environment.

### 3.3. Robustness to Positional Noise

Another type of noise present in star sensor applications is positional noise. This type of noise causes the centroid of the measured stars to shift from its actual position. This may be caused by thermal deformations of the optical system, an error of the centroiding algorithm, detector imperfections etc. In order to find the underlying robustness of the network against positional noise, an experiment was conducted that simulated centroiding error by including random pixel deviations before binning the scenes. The standard deviation for the centroiding noise was increased in steps to 1.9 pixels. The parameters used for creating the testing dataset can be found in Table 3.

**Figure 6:**
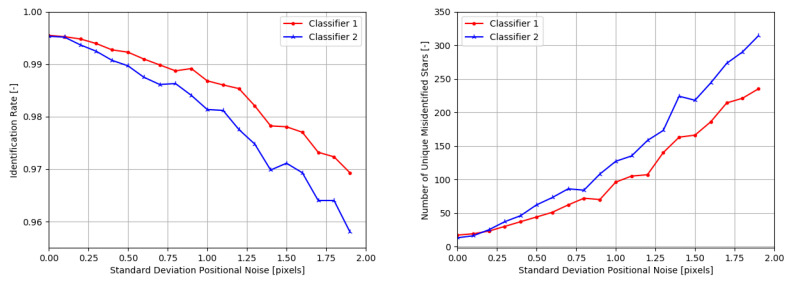
Identification rate under influence of positional noise.

The positional noise test were performed on both networks, with similar behaviour as displayed under influence of false stars. Figure 6 shows that both networks are very resilient to positional noise. Even in extreme conditions (a standard deviation of positional noise of 1.9 pixels), the identification rate is almost 97%. While the networks have not specifically been trained on this type of noise, it implicitly has learned to filter out positional noise applied directly to the image. It remains to be investigated if the network performance can be increased if the positional noise is also included in the training data.

The number of uniquely misidentified stars remains low even under challenging noise environments. For both networks, over 90% of the guide stars are always correctly identified with 1.5 pixel standard deviation in the input positional noise.

### 3.4. Sensitivity Analysis Combination of Noise Factors

By combining both the positional noise and false stars, the sensitivity of the application environment for the used configuration can be qualitatively determined. In order to find the sensitivity of the network to a combination of noise factors, 100 datasets with different noise properties were generated. The parameters of these datasets can be found in Table 4. The test was performed on the large network with 4096 nodes in both hidden layers.

**Figure 7:**
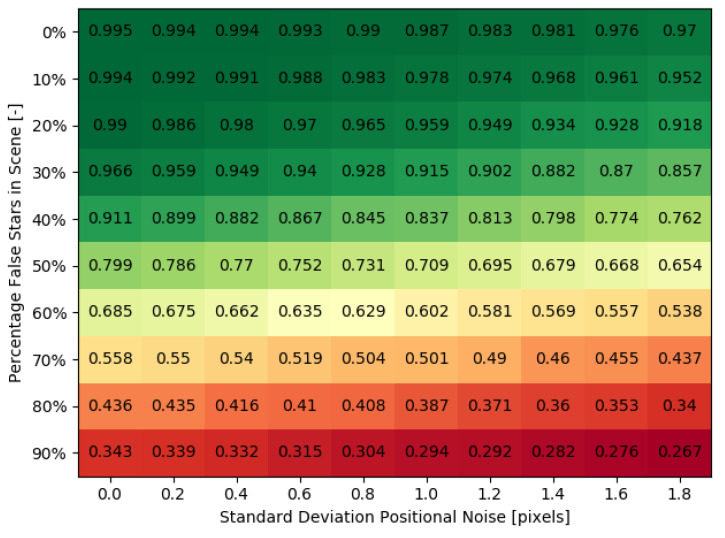
Network performance under a combination of noise factors. Numbers indicate identification rate.

Figure 7 plots the performance under influence of a combination of noise factors. The network application environment for the tested configuration has low sensitivity to the positional noise and the influence of false star is much more apparent in the performance of the network for the currently tested noise levels. However, by tuning $n_{bins}$, this behaviour can change: smaller bin sizes increases the resolution of the feature extraction, but also makes it more sensitive to positional deviations. This effect remains to be investigated.

### 3.5. Qualitative Comparison to State of the Art

Since the presented algorithm does not include a verification step at present, comparing the performance of the algorithm quantitatively to state-of-the-art algorithms would not result in representative results. However, as stated by Padgett, Kreutz-Delgado and Udomkesmalee [21] and Rijlaarsdam et al. [1] the underlying robustness of these algorithms can be compared. While our algorithm shows excellent robustness against false stars, the presented results have been achieved by generating scenes that always have a real star in the center which might not be the case in an end-to-end application. Therefore, in order to compare the underlying robustness of the algorithm it is more suitable to compare the identification rate under influence of positional noise.

Xu et al. have described the performance of their deep learning based algorithm (RPnet) [12] and that of the classical grid algorithm [2] for star identification under influence of positional noise. This performance is reported under the same FOV as the tests performed for our paper, however since the test data is not equivalent to our test data we will only deal with the underlying robustness comparison qualitatively. Remarkably, the performance of our algorithm declines at a relative constant rate as more noise is added. However, Xu et al. report nearly no performance drop up to 1 pixel standard deviation of positional nose for RPnet whereas at 1.6 pixel standard deviation of positional noise the performance of the algorithm drops to 94%. The classical grid algorithm starts to drop in performance at 0.6 pixel standard deviation of positional noise, to around 94% at 1.6 pixel [12]. However, it should be noted that this behaviour is not consistently reported for the grid algorithm across literature [1].

Therefore, at this stage of development, we conclude the following: while our algorithm has less complexity than the state of the art and provides analytically superior performance with respect to classical approaches, the underlying robustness of our algorithm against positional noise is comparable to the state of the art and appears to be better at higher levels of noise.

## 4. Discussion

The conducted experiments show that the proposed solution to the lost-in-space star identification problem is not only analytically very fast, but also robust to noise. The solution is flexible in terms of application environment: by simply increasing the width of the hidden layer, the amount of information that the network can store is easily increased. We have shown that increasing this makes the network more resilient to noise, and leave it up to the specific implementation to perform a trade-off between network size and robustness to noise.

While this experimental setup clearly shows the underlying robustness of the algorithm, it is important to note that an end-to-end star identification algorithm is also able to accept non-centralised stars as input. These polar stars should be translated to the center and the scene binned accordingly. This translation implies that the resulting patterns will be missing a portion of information, potentially reducing the performance of the neural network. This effect remains to be investigated further.

Even though the tested networks in this paper have been trained on 2306 classes, the number of accurately recognisable classes is much higher, as shown by the small decrease of performance when reducing the network size for the same number of classes. Furthermore, by increasing the number of bins, the resolution of the patterns can be dramatically increased. However, the robustness to noise might be negatively affected in this case and this trade-off remains to be investigated.

Since the network is part of an end-to-end algorithm a number of improvements for this purpose can be implemented. In future work, the non-equal bin distance should be investigated as the probability of information being present in a star scene is higher closer to the selected pole star. Another factor to consider is that the neural network will always provide an answer no matter what the input is. However, a false positive match is unacceptable for a star sensor system. Therefore it is necessary to implement a verification step in the end-to-end algorithm that employs our neural network star identification algorithm. This verification step can use the identification speed of the network to do rapid iterations for solving a scene.

Furthermore, training on more data with varying levels standard deviation and with dropped stars to simulate incomplete scenes due to translation of the pole star to the center of the pattern should be investigated, as this is likely to improve performance further.

Lastly, in future work the presented algorithm shall be implemented on dedicated hardware in order to provide relevant performance measures in terms of frames per second, power consumption etc.

## 5. Conclusions

Deep learning approaches provide analytically unrivalled performance for star identification algorithms due to the elimination of the database search. Historically both the lack of dedicated, space-graded hardware and simple and robust algorithm design have been limiting to the deployment of such algorithms. In order to address the complexity and robustness of such an algorithm, a novel feature extraction method and simple neural network design were presented in this paper. This implementation was tested on different noise environments and shown to have high underlying robustness. Since the feature extraction and the network are part of an end-to-end star sensor system a number of improvements remain to be investigated in future work, such as the implementation of a verification step. Nevertheless, the presented network was shown to be very lightweight and flexible: the number of nodes in the layers can be adapted to the application environment and even with narrow hidden layers the network has high performance in challenging conditions. The algorithm shows that even with a simple network design the classification of stars in the sparse lost-in-space star identification problem can be solved robustly.

## Figures and Tables

**Table 1:** Training dataset parameters.

Parameter	Value
Number of classes	2306
Number of scenes per class	360
Number of false stars per scene	0–4 (randomly added)
Number of bins	25
Resolution of detector	1000 × 1000 pixels
Field of view (FOV) of camera	20 × 20 degrees
Cut-off magnitude threshold	5.3 $M_{v}$
Standard deviation of added Gaussian vector noise	$20\times 10^{-6}$
Standard deviation of added Gaussian magnitude noise	0.01 $M_{v}$
Standard deviation of added Gaussian positional noise	0 pixels

**Table 2:** False star test dataset parameters.

Parameter	Value
Number of datasets	6
Number of stars in catalog	2306
Number of scenes per catalog star per dataset	10
Number of bins	25
Percentage of false stars in scene	Start at 0%, increased by steps of 10%
Standard deviation of added Gaussian vector noise	$20\times 10^{-6}$
Standard deviation of added Gaussian magnitude noise	0.01 $M_{v}$
Standard deviation of added Gaussian positional noise	0 pixels

**Table 3:** Positional noise test dataset parameters.

Parameter	Value
Number of datasets	20
Number of stars in catalog	2306
Number of scenes per catalog star per dataset	5
Number of bins	25
Percentage of false stars in scene	0%
Standard deviation of added Gaussian vector noise	$20\times 10^{-6}$
Standard deviation of added Gaussian magnitude noise	0.01 $M_{v}$
Standard deviation of added Gaussian positional noise	Start at 0 pixels, increased by steps of 0.1 pixels

**Table 4:** Sensitivity to combination of noise factors analysis dataset parameters.

Parameter	Value
Number of datasets	100
Number of stars in catalog	2306
Number of scenes per catalog star per dataset	5
Number of bins	25
Percentage of false stars in scene	Start at 0%, increased by steps of 10%
Standard deviation of added Gaussian vector noise	$20\times 10^{-6}$
Standard deviation of added Gaussian magnitude noise	0.01 $M_{v}$
Standard deviation of added Gaussian positional noise	Start at 0 pixels, increased by steps of 0.1 pixels
